# Increased Prevalence of Albuminuria in HIV-Infected Adults with Diabetes

**DOI:** 10.1371/journal.pone.0024610

**Published:** 2011-09-13

**Authors:** Peter S. Kim, Christian Woods, Lauren Dutcher, Patrick Georgoff, Alice Rosenberg, Jo Ann M. Mican, Jeffrey B. Kopp, Margo A. Smith, Colleen Hadigan

**Affiliations:** 1 National Institute of Allergy and Infectious Diseases, National Institutes of Health, Bethesda, Maryland, United States of America; 2 Department of Infectious Disease, Washington Hospital Center, Washington, D. C., United States of America; 3 University of Pennsylvania School of Medicine, Philadelphia, Pennsylvania, United States of America; 4 National Institute of Diabetes and Digestive and Kidney Diseases, National Institutes of Health, Bethesda, Maryland, United States of America; University of New South Wales, Australia

## Abstract

**Objective:**

HIV and type 2 diabetes are known risk factors for albuminuria, but no previous reports have characterized albuminuria in HIV-infected patients with diabetes.

**Research Design and Methods:**

We performed a cross-sectional study including 73 HIV-infected adults with type 2 diabetes, 82 HIV-infected non-diabetics, and 61 diabetic control subjects without HIV. Serum creatinine >1.5 mg/dL was exclusionary. Albuminuria was defined as urinary albumin/creatinine ratio >30 mg/g.

**Results:**

The prevalence of albuminuria was significantly increased among HIV-infected diabetics (34% vs. 13% of HIV non-diabetic vs. 16% diabetic control, p = 0.005). HIV status and diabetes remained significant predictors of albuminuria after adjusting for age, race, BMI, and blood pressure. Albumin/creatinine ratio correlated significantly with HIV viral load (r = 0.28, p = 0.0005) and HIV-infected subjects with albuminuria had significantly greater cumulative exposure to abacavir (p = 0.01). In an adjusted multivariate regression analysis of HIV-infected subjects, the diagnosis of diabetes (p = 0.003), higher HIV viral load (p = 0.03) and cumulative exposure to abacavir (p = 0.0009) were significant independent predictors of albuminuria.

**Conclusions:**

HIV and diabetes appear to have additive effects on albuminuria which is also independently associated with increased exposure to abacavir and HIV viral load. Future research on the persistence, progression and management of albuminuria in this unique at-risk population is needed.

## Introduction

Kidney disease is an important contributor to HIV/AIDS-related morbidity and mortality. As the life expectancy of HIV-infected patients increases with the use of antiretroviral therapy, chronic medical conditions such as renal failure are increasingly prevalent [1], [2], [3]. In addition, type 2 diabetes is increased among persons living with HIV and is associated with cumulative exposure to antiretroviral therapy [4], [5], [6]. Though the influences of both diabetes and HIV infection on kidney disease are well-recognized, there is very little known about kidney disease among HIV-infected individuals with type 2 diabetes.

Albuminuria is a marker of kidney damage, and is associated with increased cardiovascular disease and renal mortality and morbidity [7], [8], [9]. Prior to the widespread use of highly active antiretroviral therapy (HAART), studies identified a strikingly high prevalence of microalbuminuria (defined as urine albumin to creatinine ratio greater than 30 mg/g, but less than 300 mg/g), ranging from 19% to 30% among HIV-infected populations [10], [11]. More recent investigations of microalbuminuria in the post-HAART era found a prevalence between 8.7% and 11% among persons living with HIV [12], [13]. Albuminuria is common in type 2 diabetes in the absence of HIV. In one large longitudinal cohort of adults with type 2 diabetes, 25% of participants had microalbuminuria ten years after the diagnosis of diabetes [14]. Given the increased risk of microalbuminuria associated with both HIV and diabetes, we evaluated a cohort of HIV-infected adults with type 2 diabetes to assess the prevalence of albuminuria, as compared to control groups with type 2 diabetes without HIV-infection and HIV-infection without diabetes.

## Materials and Methods

### Subjects

Subjects were patients who attended HIV clinic at the National Institute of Allergy and Infectious Diseases (NIAID) outpatient clinic in Bethesda, MD or the Washington Hospital Center Infectious Diseases clinic in Washington, DC between March 2007 and February 2009. Potentially eligible participants were notified of the available research studies at routine clinic appointments and returned for subsequent research visits if interested. Participants were 73 HIV-infected adults with type 2 diabetes (defined by one or more of the following: documented diagnosis of type 2 diabetes, ICD9 codes 250.0–250.9, fasting plasma glucose ≥ 126 mg/dL on 2 or more occasions, and/or casual plasma glucose ≥ 200 mg/dL and the presence of symptoms; subjects with type 1 diabetes were excluded) and 82 HIV-infected adults without diabetes or hyperglycemia (fasting glucose <110 mg/dl, no random glucose >200 mg/dl in the past 2 years, no known diagnosis of diabetes and not on antihyperglycemic agents). Subjects with known current pregnancy, active opportunistic infection or malignancy, and known end stage renal disease or creatinine >1.5 mg/dL were excluded from participation. Subjects with HIV and diabetes were enrolled in a study of the accuracy of hemoglobin A1c which has been published previously [15]. Non-diabetic HIV-infected participants were enrolled in a study of albuminuria, but only baseline assessments were available for use in this analysis. Specific matching strategies were not used for HIV-infected subjects with or without diabetes. The study protocols were approved by the NIAID, the National Institute of Diabetes and Digestive and Kidney Diseases, and Medstar Research Institute institutional review boards. Participants gave written informed consent.

A physical exam and detailed medical history were completed and blood and urine samples were collected. A complete history of current and past antiretroviral therapy and current medications was recorded for all HIV-infected subjects. Measurements included serum creatinine, as well as urine albumin and creatinine. Albuminuria was defined as a urine albumin/creatinine ratio of >30 mg/g.

To obtain a non-HIV-infected control group with type 2 diabetes, data from 61 adults with type 2 diabetes were obtained from MedStar Health's electronic medical record system. Controls were selected based on availability of clinical data needed to meet eligibility criteria and were selected to match in age (+/− 5 years), sex and racial distribution to the HIV-infected subjects with diabetes. With the exception of HIV-related factors, exclusion criteria were the same as those for HIV-infected participating subjects. Control subjects were required to have type 2 diabetes, and have urine albumin and urine creatinine measurements from the same day as well as a documented serum creatinine measurement not greater than 1.5 mg/dL within the past year. Control data from the electronic medical records dated between 2007 and 2009 and included demographic characteristics, duration of diabetes, medication use, blood pressure and body mass index (BMI). Results of hemoglobin A1c and glycemia determinations for a subset of the subjects were reported previously [15].

### Measurements

Samples collected for serum creatinine and urine albumin and creatinine determinations were processed and analyzed at the NIH Department of Laboratory Medicine (Bethesda, MD). Urine albumin was determined via the Microalbumin (MALB) method, based on a particle-enhanced turbidimetric inhibition immunoassay adapted to the Dimension clinical chemistry system (Dade Behring, Neward, DE). Urine and serum creatinine measurements were determined using the CREA method on the Dimension clinical chemistry system (Dade Behring, Newark, DE).

Glomerular filtration rate (GFR) was calculated using the Cockroft-Gault equation and based on participant ideal body weight. All participants were required to have a creatinine ≤1.5 mg/dL and therefore the Cockroft-Gault was used for its performance in estimation of GFR in those with near normal kidney function.

In addition, stored serum maintained at −80°C from the 73 HIV-infected subjects with diabetes was available and homocysteine, IL-6, high sensitivity C-reactive protein (hsCRP), and cystatin C levels were measured. Cystatin C, hsCRP, and IL-6 levels were measured by standard ELISA assays (R&D Systems, Minneapolis, MN). Homocysteine was measured using a standard ELISA assay (Axis-Shield, Dundee, Scotland).

Laboratory results for the HIV-uninfected controls with diabetes were extracted from the electronic medical record and were completed at various MedStar facility laboratories using standardized procedures.

### Statistical Analysis

Clinical characteristics and demographic variables were compared between groups by analysis of variance, and, where significant, individual group comparisons were made using student's t-tests. Chi-square statistics were used for analyses of categorical variables. Univariate linear and logistic regression analyses were performed to identify relationships between clinical variables, albuminuria, and serum biomarkers. Multivariate regression analysis was completed that included age, race, sex, blood pressure, angiotensin-converting enzyme inhibitors (ACE) and angiotensin receptor blocker (ARB) use as well as BMI to account for possible influences these factors may have had on the prevalence of albuminuria. In a sub-analysis of only HIV-infected participants, univariate and multivariate analyses were completed to assess the relationship between HIV specific characteristics (e.g. CD4+ T-cell count, HIV viral load and antiretroviral exposure) and albuminuria. These multivariate analyses also included age, sex and race. All antiretroviral agents used by 10 or more participants were included in analyses. Non-normally distributed variables (e.g. urinary albumin/creatinine ratio) were log transformed to approximate a normal distribution. A two-tailed alpha <0.05 was used to determine statistical significance. All values are presented as mean (standard error of the mean, SEM) unless otherwise indicated. Data were analyzed using SAS JMP Version 7.0 (SAS Cary, NC).

### Results

Demographic and clinical characteristics of the 3 study groups are summarized in Table 1. Participants with diabetes (with or without HIV infection) were older, had higher BMI, were more likely to be on an ACE or ARB and were more often African American than the HIV-infected subjects without diabetes. The HIV-infected subjects with diabetes had higher mean systolic blood pressure than HIV-infected participants without diabetes, but diastolic blood pressure did not differ between groups. Serum creatinine and GFR were similar between all three study groups.

**Table 1:** Demographic and Clinical Characteristics of Subjects with HIV, Diabetes and both HIV and Diabetes.

	HIV+/Diabetes	HIV+	Diabetes	p-value
N	73	82	61	
Age (yrs)	52±1	45±1	51±1	<0.0001^a^0.9^b^<0.0001^c^
Race/ethnicity (%)				0.0002
African American	74	38	67	
Caucasian	18	48	25	
Hispanic	5	7	8	
Asian	1	6	0	
Other	2	1	0	
Sex (%)				0.1
% Male	63	78	67	
% Female	37	22	33	
BMI (kg/m^2^)	31±1	26±1	35±1	<0.0001^a^0.001^b^<0.0001^c^
Systolic BP (mmHg)	131±2	123±1	127±3	0.002 a0.16 b0.13 c
Diastolic BP (mmHg)	80±1	77±1	77±1	0.18
ACE or ARB use (%)	49%	20%	64%	<0.0001^a^0.09^b^<0.0001^c^
Duration diabetes (yrs)	6.8±0.7		6.2±0.8†	0.53
Current insulin use (%)	29		21	0.32
Hemoglobin A1c	7.1±0.2		7.8±0.3	0.03
Duration HIV (yrs)	13±1	14±1		0.38
CD4 (cells/mL)	588±32	522±30		0.13
HIV VL <50 copies/mL (%)	56	81		0.0007
Current use ARV therapy (%)	77	94		0.002
ARV therapy naïve (%)	15	1		0.0006
Duration ARV therapy (yrs)	7.7±0.6	7.9±0.5		0.8
Serum creatinine (mg/dL)	0.96±0.02	0.98±0.02	0.94±0.02	0.45
Glomerular Filtration Rate (mL/min)	82.0±2.3	88.1±2.2	82.6±1.9	0.09
Urinary albumin/creatinine ratio (mg/g)	117.5±36.8	17.7±5.4	59.9±32.2	<0.0001^a^0.1^b^0.04^c^

† data available on 43 subjects,

a – p-value for HIV+ diabetes+ versus HIV+.

b – p value for HIV+ diabetes+ versus diabetes+.

c – p value for HIV+ versus diabetes+.

Among the HIV-infected subjects, there was no significant difference in mean duration of HIV, CD4 T cell count, or duration of ARV therapy, however, those with diabetes and HIV were more likely to be ARV naive (15% vs. 1%, p = 0.0006), less likely to be on ARVs currently (77% vs 94%, p = 0.002), and less likely to have an HIV viral load <50 copies/ml (56% vs 81%, p = 0.0007). Among the subject groups with diabetes, there was no difference between those with and those without HIV infection in duration of diabetes or the use of insulin therapy. HIV-infected subjects with diabetes had a lower hemoglobin A1c compared to subjects with diabetes without HIV (7.1±0.2 vs 7.8±0.3, p = 0.03) however, we have previously reported hemoglobin A1c tends to underestimate glycemia in HIV-infected subjects [15].

**Figure 1:**
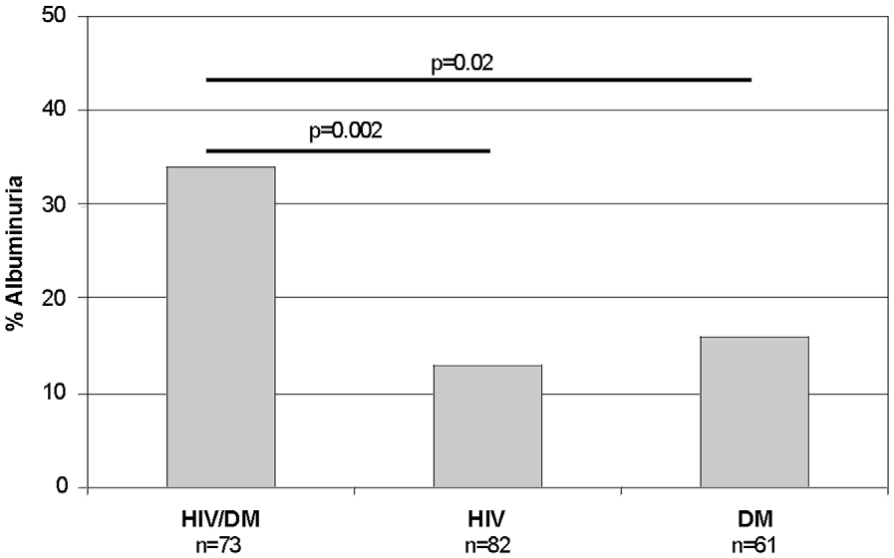
Prevalence of microalbuminuria in HIV-infected subjects with diabetes (HIV/DM), HIV-infected subjects without diabetes (HIV) and subjects with diabetes without HIV infection (DM). P values represent pairwise comparisons by chi-square.

Albuminuria was detected in 34% (25/73) of the HIV-infected subjects with diabetes, compared to 13% (11/82) in the HIV-infected non-diabetic group and 16% (10/61) in the group with diabetes and no HIV (overall χ^2^ = 10.8, p = 0.005, Figure 1). Macroalbuminuria (urine albumin/creatinine ratio >300 mg/g) was relatively uncommon and noted in only 6 HIV-infected subjects with diabetes, 1 HIV-infected subject without diabetes and 3 subjects in the diabetes alone group (p = 0.09). In univariate regression analyses, urinary albumin/creatinine ratio increased with increasing age (r = 0.19, p = 0.005) and increasing blood pressure (systolic r = 0.30, p<0.0001 and diastolic r = 0.28, p<0.0001). Among those with a diagnosis of type 2 diabetes, hemoglobin A1c was significantly correlated with albumin/creatinine ratio (r = 0.22, p = 0.01) and this remained significant in the sub-group of HIV-infected subjects with diabetes (r = 0.30, p = 0.006). The presence of albuminuria did not differ by race (χ^2^ = 4.5, p = 0.34) or sex (χ^2^ = 1.1, p = 0.3). In a multivariate logistic regression analysis, including age, race, sex, BMI, ACE/ARB use and blood pressure, both HIV infection status (p = 0.02) and diabetes status (p = 0.03) remained statistically significantly associated with the presence of albuminuria. The urinary albumin/creatinine ratio was also significantly increased in both groups with diabetes compared to the HIV-infected subjects without diabetes (Table 1).

### Biomarkers of Inflammation and Kidney Function

We determined homocysteine, cystatin C, IL-6 and high sensitivity CRP levels in the subjects with HIV infection and diabetes to evaluate whether these markers were indicative of albuminuria in this at risk population. There was no significant correlation between the biomarkers and the urinary albumin/creatinine ratio, and similarly, there was no difference in the mean levels of each biomarker in those with and without albuminuria (data not shown). There was a significant inverse correlation between cystatin C and GFR (r = −0.52, p<0.0001). BMI was also positively correlated with hsCRP (r = 0.28, p = 0.02) but there was no significant association between BMI and urinary albumin/creatinine ratio.

### HIV and Antiretroviral Therapy in Association with albuminuria

In order to evaluate potential associations between HIV specific characteristics and albuminuria, we completed a sub-analysis of all HIV-infected subjects. There was no difference in CD4+ T-cell count, duration of HIV, or total years of ARV exposure between those with and those without albuminuria (Table 2). The urinary albumin/creatinine ratio correlated significantly with HIV viral load (r = 0.28, p = 0.0005). There was no significant interaction between HIV viral load and diabetes status with regard to urinary albumin/creatinine ratio in the HIV-infected subjects. HIV-infected subjects on an ARV regimen containing abacavir were more likely to have albuminuria (% albuminuria for those on abacavir 37% vs. 19% in those not on abacavir, p = 0.03). There were no other significant differences in albuminuria by current ARV use.

**Table 2:** Comparison of HIV-Related Characteristics and Cumulative ARV Exposure in HIV-infected Subjects with and without Albuminuria.

	Albuminuria (n = 36)	No Albuminuria (n = 119)	p-value
Duration HIV (y)	13.1±1.0	13.7±0.6	0.6
Duration ARV exposure (y)	7.4±0.9	7.9±0.4	0.6
CD4 T cell count	498±42	570±26	0.15
HIV viral load (log copies/ml)	2.55±0.2	2.10±0.08	0.046
**Cumulative NRTI Exposure**			
Abacavir (mos)	30±7	11±2	0.01
Didanosine (mos)	15±6	11±2	0.6
Emtricitabine (mos)	8±2	12±2	0.09
Lamivudine (mos)	53±8	63±4	0.3
Stavudine (mos)	21±6	21±3	0.99
Tenofovir (mos)	19±4	22±2	0.5
Zalcitabine (mos)	4±2	1±0.2	0.2
Zidovudine (mos)	30±6	42±4	0.09
**Cumulative PI Exposure**			
Atazanavir (mos)	7±2	8±1	0.6
Indinavir (mos)	6±3	15±3	0.01
Lopinavir (mos)	13±4	14±2	0.8
Nelfinavir (mos)	13±5	12±2	0.9
Ritonavir (mos)	19±5	16±2	0.6
Saquinavir (mos)	5±2	3±1	0.5
**Cumulative NNRTI Exposure**			
Efavarenz (mos)	19±5	22±3	0.6
Nevirapine (mos)	12±5	8±2	0.5

HIV-infected subjects with albuminuria had significantly greater cumulative exposure to abacavir (30.4 mos vs. 10.9 mos, p = 0.01) and less exposure to indinavir (5.8 mos vs. 15.2 mos, p = 0.02) compared to those without albuminuria. Cumulative exposure of other antiretroviral agents, including tenofovir, did not differ by albuminuria. These observations were replicated in univariate regression analysis of cumulative duration of specific ARV exposures and urinary albumin/creatinine ratio, where only abacavir and indinavir showed significant relationships to albumin/creatinine ratio (abacavir mos r = 0.19, p = 0.02; indinavir mos r = −0.17, p = 0.03). In a multivariate logistic regression analysis which included age, race, sex, BMI, blood pressure, and ACE/ARB use, the diagnosis of diabetes (p = 0.003), higher HIV viral load (p = 0.04) and cumulative exposure to abacavir (p = 0.001) were all significant independent predictors of albuminuria in HIV-infected subjects. Indinavir exposure was no longer significant (p = 0.08). The association between albuminuria and abacavir (p = 0.002) and the diagnosis of diabetes (p = 0.003) remained significant after further adjustment for total years of ARV exposure. Each of the statistical analyses was repeated excluding the 10 subjects with macroalbuminuria, and there were no changes in statistical significance with regard to the major findings (data not shown).

## Discussion

Though albuminuria is considered an important complication of both diabetes and HIV, our study is the first to report on the prevalence of albuminuria among individuals affected by both diseases. We found that HIV and diabetes have additive effects in regards to early kidney dysfunction. This was demonstrated by the presence of albuminuria identified in 34% of HIV-infected diabetic subjects compared to control groups with HIV alone (13%) or diabetes alone (16%).

In the present study, the effects of HIV and diabetes on albuminuria were significant even after the effects of age, race, BMI, blood pressure and ACE/ARB use, factors known to influence albuminuria, were taken into account. The prevalence of albuminuria among the HIV-infected subjects without diabetes was similar to the 11% identified by Szczech and colleagues in a similar HIV-infected cohort [13]. The prevalence of albuminuria noted among the uninfected controls with diabetes was slightly lower than other large diabetes cohort studies [14], [16], but the duration of diabetes in our study population was relatively short by comparison and may account for this difference. We did not identify an increased prevalence of albuminuria related to African American race or Hispanic ethnicity as seen in prior large US population surveys [17]. The percentage of African American subjects was highest among the groups with diabetes and therefore it may have been difficult to identify independent influences based on race. Among those participants with HIV, we did not find a relationship between albuminuria and CD4+ T-cell count; however, there was a positive correlation between HIV viral load and levels of albuminuria. The increased prevalence of HIV viremia among the HIV-infected subjects with diabetes may have contributed, at least in part, to the high prevalence of albuminuria observed in this group. These data suggest that active HIV infection may contribute to a broad spectrum of kidney injury, ranging from microalbuminuria to more severe injury such as HIV-associated nephropathy [18]. Alternatively, viremia may be a marker of medication non-adherence which itself may play a role in albuminuria in individuals with HIV and diabetes.

Glycemic control is one potential contributor to microalbuminuria in the setting of diabetes. Despite the lower HbA1c levels noted in the HIV-infected subjects with diabetes compared to controls with diabetes alone, we and others have shown that HbA1c tends to underestimates glycemia in HIV[15], [19]. Further, HIV-infected participants with diabetes were less likely to be on an ACE or ARB medication compared to participants with diabetes alone. Therefore, practice disparities in health management of glycemic control and blood pressure in the setting of HIV and diabetes may contribute to the increased rates of microalbuminuria observed in this group.

Of interest, we found that HIV-infected subjects with albuminuria had significantly greater cumulative exposure to abacavir and less exposure to indinavir when compared to those without albuminuria. Analysis of exposure to other ARV agents did not show significance in this regard. The inverse association between indinavir and albuminuria may be secondary to confounding. Namely, clinicians may have been disinclined to prescribe or may have discontinued indinavir for patients with perceived risk of kidney disease due to the known association between indinavir use and urinary crystal formation. Similarly, one might argue that increased abacavir exposure in those with albuminuria was observed due to confounding in prescribing practices. For example, clinicians may have preferentially used abacavir rather than tenofovir in individuals with risk factors for kidney disease. However the present data do not support this consideration because cumulative, current and any exposure to tenofovir did not differ between those with albuminuria and those without.

Recent use of abacavir has been implicated in chronic kidney disease[20]. Here we noted increased albuminuria in association with both current and cumulative abacavir exposure, which may represent a relationship between abacavir and early endothelial injury. Recent large cohort studies and randomized trials have implicated abacavir in the development of cardiovascular disease in HIV-infected adults [21], [22]. However chronic kidney disease is also linked to cardiovascular disease events in HIV, further complicating the association between abacavir and vascular events [23].

Microalbuminuria is often considered a harbinger of more significant vascular disease; therefore the current observation linking albuminuria to abacavir exposure may represent endothelial injury through as-yet-undefined mechanisms. Investigation by Hsue and colleagues found that current abacavir use was independently associated with impaired endothelial function as measured by flow mediated vasodilation [24]. This effect was not readily explained by traditional cardiovascular disease risk factors, nor was it related to other HIV specific measures such as CD4+ T-cell count and duration of ARV therapy.

Studies of type 2 diabetes, in the absence of HIV infection, have observed associations between inflammatory markers such as IL-6 and CRP and degree of albuminuria [25], [26], [27]. In the present study we did not identify significant correlations between hsCRP or IL-6 and albuminuria among HIV-infected diabetic subjects. The relatively small sample size may limit our ability to detect such relationships. Alternatively, non-inflammatory processes such as medication effects and direct viral toxicity may be stronger mediating factors of albuminuria in this unique population. Further, 30% of the HIV-infected diabetic subjects had hsCRP levels above the limit of the assay (>5.0 microgram/ml), indicating a relatively high level of inflammation and possibly limiting our ability to detect linear relationships between hsCRP and other variables.

Interpretation of the current findings is limited due to the cross-sectional nature of the study and therefore identified associations cannot be used to assign causality. There is also a possibility for selection bias in that participants concerned with diabetes and or kidney function may have been more likely to agree to participate. In addition, we performed a single measure of urinary albumin and creatinine levels, whereas multiple determinations would provide a more accurate estimate of the true prevalence of albuminuria in this population [12]. Currently, one of the largest studies estimating the prevalence of microalbuminuria in HIV also used a single determination [13] and the prevalence estimate of 11% was similar to the 8.7% found in a recent HIV study which utilized 3 urine measurements [12].

In summary, we found that the prevalence of albuminuria in individuals with HIV and diabetes was two fold greater than that of individuals with either disease alone. The albuminuria was associated with traditional risk factors such as increasing age and blood pressure, poor glycemic control, but was also independently associated with increased exposure to abacavir and HIV viral load. Though screening for microalbuminuria and recommendations for its management are well-defined for diabetic patients, there are no recommendations specific to HIV. High quality studies that obtain serial determinations of urinary albumin/creatinine ratios in HIV-infected patients both with and without diabetes are needed to better estimate the prevalence of this problem. The high estimate of albuminuria among HIV-infected, diabetic subjects observed with a single determination in this study is concerning and further research on the persistence, progression and management of albuminuria in this unique at-risk population is needed.
